# Phenolic Compounds as Unambiguous Chemical Markers for the Identification of Keystone Plant Species in the Bale Mountains, Ethiopia

**DOI:** 10.3390/plants8070228

**Published:** 2019-07-16

**Authors:** Bruk Lemma, Claudius Grehl, Michael Zech, Betelhem Mekonnen, Wolfgang Zech, Sileshi Nemomissa, Tamrat Bekele, Bruno Glaser

**Affiliations:** 1 Institute of Agronomy and Nutritional Sciences, Soil Biogeochemistry, Martin Luther University Halle–Wittenberg, Von–Seckendorff–Platz 3, D–06120 Halle, Germany; 2 Ethiopian Biodiversity Institute, Forest and Rangeland Biodiversity Directorate, P.O. Box 30726 Addis Ababa, Ethiopia; 3 Institute of Computer Science, Bioinformatics, Martin Luther University Halle–Wittenberg, Von Seckendorff-Platz 1, 06120 Halle (Saale), Germany; 4 Institute of Geography, Technical University of Dresden, Helmholtzstrasse 10, D–01062 Dresden, Germany; 5 Department of Urban Agriculture, Misrak Polytechnic College, P.O. Box 785, Addis Ababa, Ethiopia; 6 Institute of Soil Science and Soil Geography, University of Bayreuth, Universitätsstrasse 30, D–95440 Bayreuth, Germany; 7 Department of Plant Biology and Biodiversity Management, Addis Ababa University, P.O. Box 3434 Addis Ababa, Ethiopia

**Keywords:** paleoclimate, pollen, paleovegetation, oxidation, phenols, *Erica*, biomarkers, machine learning

## Abstract

Despite the fact that the vegetation pattern and history of the Bale Mountains in Ethiopia were reconstructed using pollen, little is known about the former extent of *Erica* species. The main objective of the present study is to identify unambiguous chemical proxies from plant-derived phenolic compounds to characterize *Erica* and other keystone species. Mild alkaline CuO oxidation has been used to extract sixteen phenolic compounds. After removal of undesired impurities, individual phenols were separated by gas chromatography and were detected by mass spectrometry. While conventional phenol ratios such as syringyl vs. vanillyl and cinnamyl vs. vanillyl and hierarchical cluster analysis of phenols failed for unambiguous *Erica* identification, the relative abundance of coumaryl phenols (>0.20) and benzoic acids (0.05—0.12) can be used as a proxy to distinguish *Erica* from other plant species. Moreover, a Random Forest decision tree based on syringyl phenols, benzoic acids (>0.06), coumaryl phenols (<0.21), hydroxybenzoic acids, and vanillyl phenols (>0.3) could be established for unambiguous *Erica* identification. In conclusion, serious caution should be given before interpreting this calibration study in paleovegetation reconstruction in respect of degradation and underground inputs of soil organic matter.

## 1. Introduction

The present vegetation cover in mountain ecosystems is the result of subsequent historical and ecological processes primarily influenced by climate and human [1,2]. However, natural versus human impact on ecosystem dynamics at higher elevation is difficult to quantify and is still speculative [1,3]. To reconstruct and draw full pictures of environmental changes over the past centuries, Ethiopia is an ideal place for several reasons. Firstly, it is the origin of *Homo sapiens* and many other fauna and flora. Secondly, it comprises diverse geographical zones attributed to various climatic features [4,5]. The Bale Mountains are one of the Eastern Afromontane Biodiversity Hotspots, which encompass many endemic and endangered species of Ethiopia [6,7]. Diverse topographical features of the Bale Mountains are marked by altitudinal vegetation zones. The ecosystem of the Bale Mountains is divided into three main declivities and five distinct and unique habitats. The Southern declivity is demarcated by moist Afromontane Harenna forest and a prominent Ericaceous belt whereas, the central plateau is dominated by the Afro-alpine vegetation, and the Northern slopes encompass dry Afromontane forest, as well as Gaysay grassland [6,8,9]. The afro-alpine regions of the Sanetti Plateau (3800–4377 m a.s.l.) are characterized by tussock grasses (mainly *Festuca* spp.), dwarf shrubs, and herbaceous plants (e.g. *Alchemilla haumanii*, *Helichrysum splendidum* and *Lobelia rhynchopetalum*) [8,9,10]. The prominent Ericaceous belt dominant above 3300 m a.s.l shows different stages of post-fire succession and remains continuous up to 3800 m a.s.l. However, it becomes patchy on the Sanetti Plateau above 3800 m a.s.l. [9]. The characteristic species of the Northern slopes are *Hagenia abyssinica*, *Hypericum revolutum*, *Juniperus procera,* and *Podocarpus falcatus* [6,8].

In the Bale Mountains, the contemporary distribution of the *Erica* vegetation in specific and of the afro-alpine biodiversity in general is the legacy of climate change [2] or human-induced driving factors like fire and grazing [9] or a combination of both. Hitherto, this globally important ecosystem is still increasingly under threat of climate change and human impacts [11]. The vegetation history of the Bale Mountains was investigated and reconstructed using pollen records from lacustrine sediments and peat deposits [2,12,13,14]. The findings showed that *Erica* shrubs inhabited different altitudinal zones during different time periods. During the early Holocene, *Erica* shrubs had extended up to the Sanetti plateau in response to increased moisture and temperature [2]. *Erica* shrubs and forest had retreated to lower altitude in response to lower rainfall at about 6000 cal years BP and onwards [2]. Presently, *Erica* forming an Ericaceous belt is located between 3300 to 3800 m. a.s.l. [9].

The biggest deterrent of pollen-based studies depends on pollen preservation and can be biased by the dispersal potential of pollinators [3,15,16]. To overcome such drawbacks, we tested, for the first time, the chemotaxonomic roles of phenol biomarkers in the dominant plant species of the Bale Mountains. Chemical biomarkers are not influenced by a dispersal of parent material and can be extracted from sedimentary archives and soils and reflect more of the standing vegetation [17]. Therefore, they offer the potential to complement pollen-based vegetation reconstructions and to detect vegetation at a higher temporal and spatial resolution.

Polyphenols are the most widespread class of secondary metabolites, and ubiquitous in their distribution [18]. Phenolic compounds are a major component of terrestrial vascular plants [19] and may have lignin and non-lignin-derived biochemical structures. Secondary metabolites, mainly phenolic compounds, are important for cell wall structural formation, defense against pathogen, and environmental stress as well as essential for survival of vascular plants [20,21]. Plant phenolic compounds also exhibited significant qualitative and quantitative variation at different genetic level between and within species and clones [22,23,24]. They are extensively used in botanical chemosystematic studies [20]. The chemotaxonomic values of polyphenolic biomolecules such as tannins has been well recognized in the plant kingdom and the distribution of hydrolysable tannins used as chemotaxonomic markers in the family *Rosaceae* [25,26] and chestnut (family: *Fagaceae*) [27]. Stilbenoids, family of polyphenols, due to its limited but heterogeneous distribution in the plant kingdom also played a significant taxonomic role in the plant families such as *Vitaceae*, *Gnetaceae*, and *Fabaceae* [28]. Notably gossypetin (flavonol) are useful taxonomic marker for distinguishing Ericaceous species/genera (family: *Ericaceae*) [29]. The absence of leucoanthocyanidins, ellagic acid and phenyl-trihydroxylated characterized the genus *Pittosporum* (Pittosporaceae) [30]. Even if there is a need to encourage phenols application in chemotaxonomic studies/plant phylogeny, as more studies have been completed, some inconsistence has been revealed [31]. For instance the polyphenolic compound differences among the various tribes of *Anacardiaceae* are not very pronounced [32].

Nevertheless, little has been done to chemotaxonomically characterize *Erica* and other keystone species specifically in the Bale Mountains and generally in other similar geographical regions using phenols as a tool for paleovegetation reconstruction. Apart from the chemotaxonomic significance of phenols, the antioxidant and antibacterial activities of phenolic compounds by Guendouze-Bouchefa et al. [33], as well as the role of phenolic compounds as indicators of presence of metals in the leaves of different genera in the family *Ericaceae* by Marquez-Garcia et al. [34,35] were described previously. The antioxidant strength of *Erica* species depend upon the total abundance of phenolic compounds [34]. The abundance of phenolic compounds in *Festuca vallesiaca* were also used as indictors of fodder quality. High contents of total phenolic in the grass species are unfavorable for digestion [36]. Polyphenols obtained from flowers of *Helichrysum* (family: *Asteraceae*) species are used by some folks as ethno-medicine [37,38,39,40]. The affinity bounds in plant systematic classification using profiles of phenolic compounds were computed and compared, commonly using hierarchal cluster analysis (HCA) and principal component analysis (PCA) [20,41,42,43].

While the overall aim of our research is to understand how humans benefited from and re-shaped the African high altitude ecosystem during Quaternary climate changes, this study mainly focuses on identifying the potential of plant derived phenolic compounds as a proxy for unambiguous identification of *Erica* and other keystone species in the Bale Mountains. More specifically, this research attempts to addresses the following questions: (i) Do phenol biomarkers allow a chemotaxonomic differentiation of the contemporary dominant plant species of the Bale Mountains? (ii) Which implications have to be drawn from those results for planned paleovegetation reconstructions in the study area, e.g., concerning the reconstruction of the former extent of *Erica* stands?

## 2. Results and Discussion

### 2.1. Distribution and Diversity of Phenols

**Figure 1:**
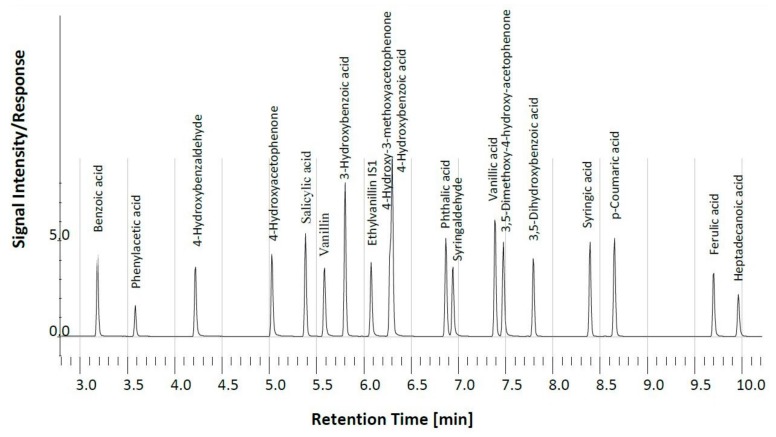
Typical gas chromatogram of phenolic compounds released after alkaline CuO oxidation of *Lobelia rhynchopetalum*.

A typical gas chromatogram of phenolic compounds released after mild alkaline CuO oxidation illustrates the compositional complexity of a typical plant sample (*Lobelia rhynchopetalum*; Figure 1). The mean weighted sum of phenols content of *Alchemilla haumanii*, *Erica* spp., *Helichrysum splendidum*, *Kniphofia foliosa*, *Lobelia rhynchopetalum,* and *Festuca abyssinica* were 18, 16, 22, 6, 22, and 51 g kg^−1^ TOC, respectively (Table S1).

**Figure 2:**
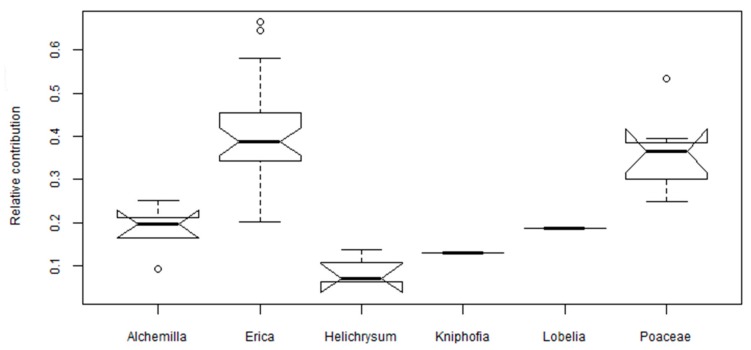
Notched box plots illustrating the abundance of coumaryl phenols in the dominant plant species of the Bale Mountains. The box plots indicate the median (solid line between the boxes), interquartile range (IQR) with upper (75%) and lower (25%) quartiles and possible outlier (white circles). The notches display the confidence interval around the median within +/−1.57 * IQR/√n.

*Festuca abyssinica* (Poaceae) is characterized by high abundance of coumaryl phenols (*p*–coumaric acid, ferulic acid) and syringyl phenols (syringic acid, 3,5-Dimethoxy-4-hydroxyacetophenone; Figure 2) and low abundance of vanillyl phenols and benzoic acids (Table 1). Similarly, the phenolic compounds from *Festuca vallesiaca* species investigated in Serbia accounts for low benzoic acid derivatives and high coumaryl phenols (*p*–coumaric acid, ferulic acid). The total phenolics extracted from *Festuca vallesiaca* using Soxlet extraction and later quantified by HPLC is 26.1 mg g^−1^ [36]. In the grass taxon, most of phenol-related information was provided by coumaryl phenols (*p*–coumaric acid, ferulic acid) due to ample presence of the enzyme phenyl (thyrosine) ammonia-lyase and it is known for its high biological activity [20,36,44]. The abundance of coumaryl phenols in our *Poaceae* samples holds one third of the total phenols. Likewise, Hedges and Mann [45] stated that non woody vascular angiosperm plants have characteristic products of coumaryl phenols and it accounts for approximately one third of the total phenolic compounds. *Lobelia rhynchopetalum* and *Kniphofia foliosa* have high abundance of benzoic acid and 4-hydroxy-3-methoxyacetophenone (Table 1). The total phenolic contents of *Lobelia chinensis* measured by the Folin-Ciocalteu methods is 4.7 mg GAE g^−1^ [46].

**Figure 3:**
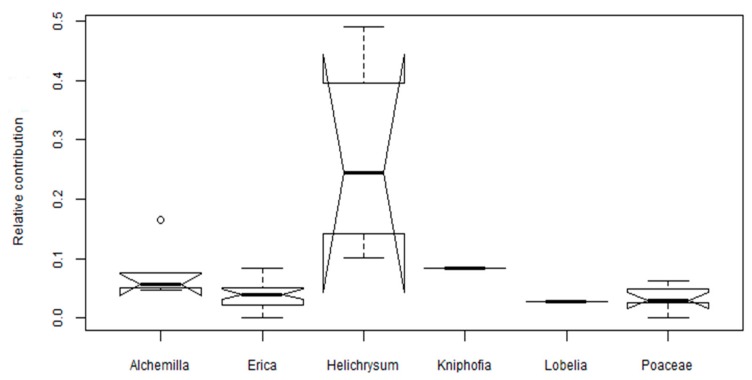
Notched box plots show the relative abundance of *p*-hydroxy phenols in the dominant plant species of the Bale Mountains. The box plots indicate the median (solid line between the boxes), interquartile range (IQR) with upper (75%) and lower (25%) quartiles and possible outlier (white circles). The notches display the confidence interval around the median within +/−1.57 * IQR/√n.

The sum of *p*-hydroxy phenols in the leaves unambiguously identifies *Helichrysum splendidum* from the other dominant plant species of the Bale Mountains (Figure 3). Qualitative analysis by thin layer chromatography (TLC) of the inflorescence of *Helichrysum* showed the presence of phenolic acids such as syringic, coumaric, and *p*-hydroxybenzoic [40]. The total phenolic contents extracted from different species of *Helichrysum* in Eastern Anatolia, Turkey ranges between 72 to 146 mg GAE g^−1^ [39]. On the other hand, *Erica* species were characterized by high coumaryl (Figure 2) and low *p*-hydroxy phenols (Figure 3). The genus *Erica* (family: *Ericaceae*) is characterized by having polyphenols (*p*-coumaric acid derivate, vanillic acid, cinnamic acid derivate, and caffeic acid) and flavonoids [33]. The total phenolic compounds determined via Folin-Ciocalteu method and analysis by HPLC for *Erica arborea* and *Erica multiflora* are 71 and 68 mg GAE g^−1^, respectively [33]. In the genus *Erica* among the flavonoids, dihydromyricetin 3-*O*-α-l-rhamnopyranoside is the most important chemotaxonomic marker [47]. Each of the dominant plant species on the Bale Mountains is profiled by a different abundance of phenolic compounds (Table 1).

Even though *Kniphofia foliosa* has lower phenolic content, the quantitative values of Shannon-Wiener phenol diversity index (H = –sum (p_i_)[In(p_i_)]) indicated that *Kniphofia foliosa* has evenly distributed phenolic compounds. By contrast, *Helichrysum splendidum* has less evenly distributed phenolic compounds (Table 2). Innovative techniques and widespread occurrence of secondary metabolites like phenolic profiles in the plant diversity allow low taxonomic level chemosystematic studies [18,20].

### 2.2. Cluster Analysis of Phenolic Compounds

**Figure 4:**
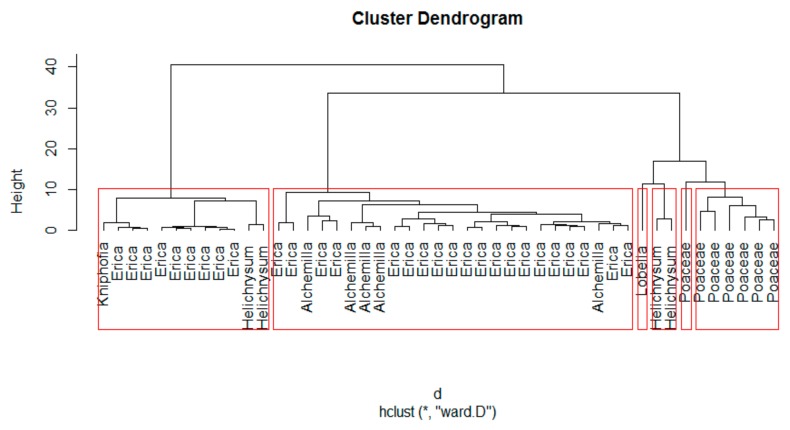
Agglomerated Hierarchical Cluster analysis of the dominant plant species of the Bale Mountains based on the abundance of phenolic compounds (g kg ^−1^ TOC). Euclidean measurement of distances and Ward method for linkage calculation applied to cluster a normalized dataset (Z-score normalization, Z = (X-mean (X))/sd (X)).

The cluster analysis computed for 47 plant samples grouped the dominant plant species into six groups, as shown in Figure 4. In the cluster, *Poaceae* and *Lobelia* species are grouped independently (there are two independent *Poaceae* groups), while the other dominant species *Erica*, *Kniphofia*, *Helichrysum*, and *Alchemilla* were clustered together into two different groups (Figure 4). Therefore, no unambiguous identification of *Erica* is possible, but identify the former extent of the distribution of *Poaceae* in the Bale Mountains using alkaline CuO phenol products might be feasible (Figure 4). Previously, some studies used phenolic compounds to systematically characterize grasses even at lower taxonomic level [18]. However, phenols are environmentally labile secondary metabolites. As a result, caution should be given before extraction and standardizing the growing condition and growth stage of the sample materials [20,21,48]. Thus far, our cluster analysis did not justify which phenol type is the most determining characteristic for the identification of the dominant plant species, one from the other. The only obvious independent group is the *Lobelia* one, and *Poaceae* is also somewhat distinct. Therefore, the ambiguity is less clear for all the rest.

### 2.3. Biomarker Identification

**Figure 5:**
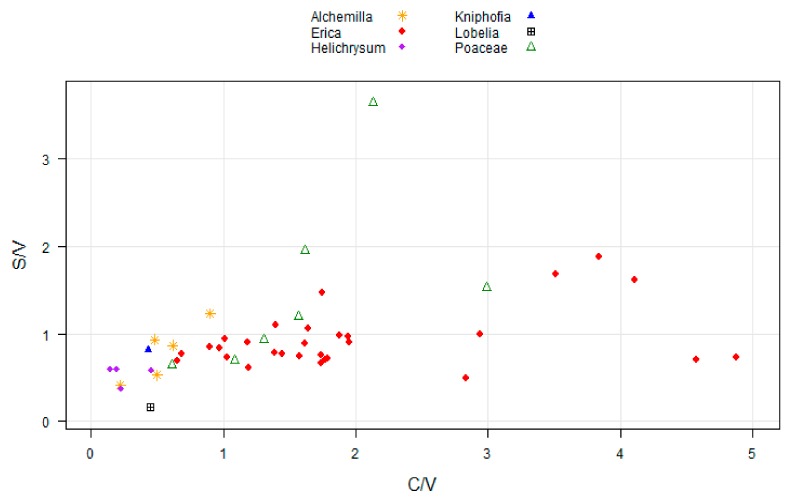
Two-dimensional plot illustrating the ratios of syringyl vs. vanillyl (S/V) and coumaryl vs. vanillyl (C/V) phenols of the dominant plant species under study.

The ratios of syringyl over vanillyl (S/V) and cinnamyl over vanillyl (C/V) phenols, which were used previously as a source proxy did not turn out to be useful in the present study to categorize the dominant plant species (Figure 5). The ratios of S/V and C/V allowed us to distinguish organic matter sources in soil and sediments [45]. However, these ratios cannot provide profound results of plants belonging to the same taxon [49]. The limitation of S/V and C/V ratios to pinpoint the source of organic matter in soils and sediments of the same taxon were described in detail by Thevenot [49]. Here, we also proved that the ratios of S/V and C/V were unable to characterize modern plants of the same taxon at least in our study area (Figure 5). The drawback of those ratios could be associated with the overlap between plant molecular signatures [49,50]. The sampled woody vegetation (e.g *Erica*, *Helichrysum*) in the present study exhibited C/V ratios higher than zero. However, Hedges and Mann [45] stated that woody gymnosperm and angiosperm plants are characterized by C/V ratio nearly equal to zero.

**Figure 6:**
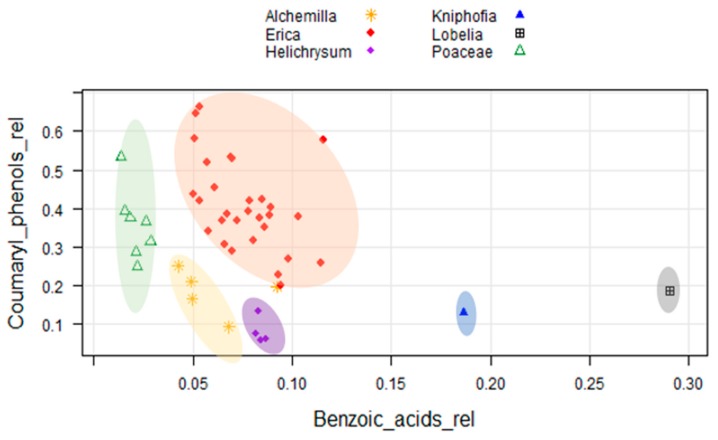
Two-dimensional plot of the relative contribution of the sum of coumaryl phenols (*p–*coumaric acid + ferulic acid) and benzoic acids (benzoic acid + salicylic acid + phthalic acid) in the dominant plant species under study.

We here evaluated the phenolic compounds via the classical approach such as hierarchal clustering, 2D plots analysis using source proxies (S/V and C/V) to chemotaxonomically characterize locally dominant plant species in the Bale Mountains. Both approaches failed to explicitly characterize the dominant plant species. However, we found that *Erica* vegetation in the Bale Mountains can be identified using the relative contribution of coumaryl phenols (>0.2) and benzoic acids (0.05–0.12; Figure 6).

**Figure 7:**
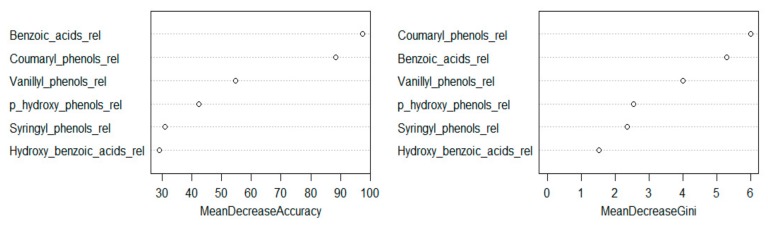
Set of relative phenols variables used for chemotaxonomy ordered by their importance as estimated by the Random Forest model. 32 training sample sets and 10.000 number of trees were used to compute the model.

**Figure 8:**
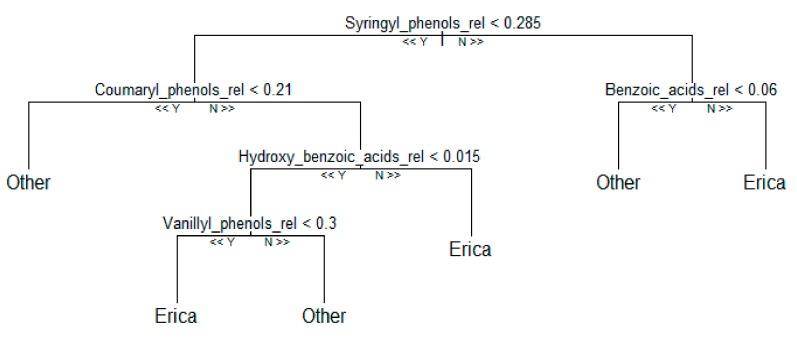
Representative decision tree of Random Forest model using relative phenols contribution. 32 training sample sets and 10,000 number of trees were used to compute the model.

Three different machine-learning algorithms namely Support Vector Machine, Random Forest, and Recursive Partitioning were tested to unambiguously identify *Erica* vegetation based on our phenolic compounds dataset. Among the tested machine learning approaches, Random Forest performed best. The proposed algorithm has been shown to be better in terms of various performance indicators like accuracy (Figure S1A) and F1-score (Figure S1B). In the experiment using relative phenols dataset, the key variable for chemotaxonomic classification of the contemporary species in the Bale Mountains are given in Figure 7. Among them, benzoic acids (<0.06) and coumaryl phenols (0.21) were the most decisive variables to identify *Erica* from the other species (Figure 6 and Figure 8). The cross-validation of the used model is shown in Figure S3.

## 3. Material and Methods

### 3.1. Study Area

#### 3.1.1. Geomorphological Setting

**Figure 9:**
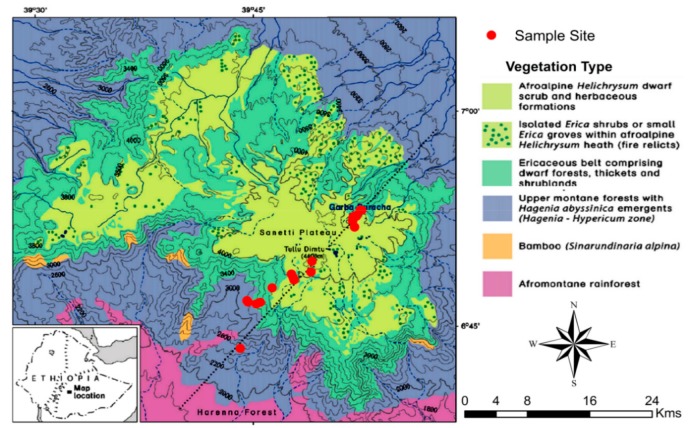
Map of the Bale Mountains showing the vegetation zones and study sites along the Northeast and Southwestern transect (modified after Miehe and Miehe, 1994). Leaves of the dominant vegetation within the Ericaceous and afro-alpine belts were sampled during the dry season in February 2015 and 2017.

The Bale Mountains belong to the Bale-Arsi mountain massif located 400 km Southeast of Addis Ababa, Ethiopia [6]. They are situated between 39°03′ to 40°00′ longitude (E) and 6°29′ to 7°10′ latitude (N) (Figure 9). Lava mainly outpouring 40 million years ago resulted in the formation of mountains and ridges up to 4377 m a.s.l. (Mohr, 1963). The most extensive high altitude plateau (Sanetti Plateau) in Africa above 3000 m. a.s.l., comprised within the Bale Mountains National Park, extends to about 2,600 km^2^ [2,5,7,11]. The Bale Mountains are topographically divided into three major declivities: The Northern slopes (3000–3800 m. a.s.l.), the central plateau (3800–4377 m a.s.l), and the Southern Harenna escarpment (1400–3800 m. a.s.l.). The Sanetti Plateau is located in the center, on which the second highest peak of the country Mt. Tulu Dimtu (4377 m a.s.l.) rests. The northern slope of the mountains is dissected by the Togona Valley, which descends gently towards the extensive Arsi Plateau and further down to the Great Rift Valley lowlands, which divide the country into two parts. The Southern slope includes the steep Harenna escarpment and goes down to the surrounding lowland at about 1400 m a.s.l. [5,7]. Repeated glaciations of the high altitudes created typical features of glaciated landscapes with moraines and glacial lake [2,51]. Basalt and rhyolite are typical parent rocks [52] of the dominant Cambisols and Leptosol at the Sanetti Plateau [9,10,53].

#### 3.1.2. Climate and Biota

The climatic conditions of the Bale Mountains depend on orography and are vulnerable to extreme climatic conditions over the past years [2]. The mean annual minimum temperature in the mountainous region ranges between 0.6 to 10 °C with frequent frost during winter season and mean annual maximum temperature ranges between 6–12 °C. The mean annual temperature in Dinsho headquarter (3170 m a.s.l.) is 11.8 °C [5,54]. Precipitation in the Bale Mountains is influenced by the shift of the Inter Tropical Convergence Zone (ITCZ) resulting in long rainy (March–September) and short dry (October–February) seasons [55]. The Southern slopes of the Bale Mountains receive high annual rainfall (1000–1500 mm year^−1^) as compared to the Northern declivity (800–1000 mm year^–1^) [5,53,54]. Moisture reaching the Bale Mountains originates from the Red Sea, the Mediterranean Sea, the Indian Ocean, and the Atlantic Ocean [9,56,57].

The Bale Mountains National Park and its surrounding are home for more than 1300 vascular flowering plant species and 50 mammal species [7,58]. The composition of enormous endemic and endangered fauna and flora labeled the Bale Mountains as one of the biodiversity hotspot areas of the world. The distribution and abundance of the contemporary vegetation in the Bale Mountains are shaped partly by human intervention and often emphasizes the landforms and paleoclimate [51]. Diverse topographical variability in the Bale Mountains shows altitudinal zonation of vegetation. In broad terms, floristically the mountains are divided into three topographical regions. The southern declivity is delineated by moist Afromontane forest, Ericaceous belt and Afro-alpine vegetation, whereas the central plateau is dominated by the Afro-alpine vegetation and the northern slope encompasses dry Afromontane forest, Gaysay grassland, Ericaceous belt, and afro-alpine zones [6,8,9]. The afro-alpine regions of the Sanetti Plateau (3800–4377 m a.s.l.) are characterized by tussock grassland and dwarf shrubs and herbaceous (e.g., *Alchemilla haumanii*, *Helichrysum splendidum,* and *Lobelia rhynchopetalum*) plants. The Ericaceous belt (3300–3800 m a.s.l.) comprises forest, thickets, and scrublands of *Erica trimera* and *Erica arborea* with mosses and grasses dominating in the ground layer. The Harenna forest (1500–3300) is a natural remnant of a moist Afromontane forest dominated by broadleaved evergreen trees and clustered floristically into two different classes. Around 1500–2300 m a.s.l., the forest is dominated by *Podocarpus falcatus* associated with *Croton macrostachyus*, *Pouteria adolfi-friederici*, *Syzygium guineense*, *Warburgia ugandensis,* and worth noting *Coffea arabica*. Between 2300–3200 m a.s.l. *Arundinaria alpina*, *Hagenia abyssinica*, *Hypericum revolutum*, *Erythrina brucei*, *Prunus africana,* and *Schefflera volkensii* are the dominant plants [2,7,8,9,53]. Furthermore, the Bale Mountains are center for faunal diversity and holds the highest rate of animal endemicity for a terrestrial habitat anywhere in the world [7]. The mountains are known by its flagship mammals like Mountain Nyala (*Tragelaphus buxtoni*), Ethiopian Wolf (*Canis simensis*) and Giant mole rat (*Tachyoryctes macrocephalus*). Also about 180 bird species and 14 amphibian species are inhabiting the Bale Mountains [6,7].

### 3.2. Sample Collection

25 and 22 (total of 47) samples (leaves and twig) of locally dominant plant species were collected in February 2015 and 2017, respectively along a SW and a NE transect (ranging from 2550 to 4377 m a.s.l. and 3870 to 4134 m a.s.l., respectively) (Figure 9). Samples comprise *Erica trimera* and *Erica arborea* (*n* = 29), *Alchemilla haumannii* Rothm. (*n* = 5), *Festuca abyssinica* Hochst. ex A. Rich. (*n* = 7)*, Helichrysum splendidum (Thunb.) Less.* (*n* = 4), *Kniphofia foliosa Hochst.* (*n* = 1) *and Lobelia rhynchopetalum* Hemsl. (*n* = 1). All samples were air-dried in the Soil Store Laboratory of the National Herbarium, Department of Plant Biology and Biodiversity Management, Addis Ababa University. Samples were finely grinded, homogenized, and subjected to further biogeochemical analysis at the laboratories of the Soil Biogeochemistry, Martin Luther University of Halle–Wittenberg.

### 3.3. Analysis of Phenolic Compounds Released after Alkaline CuO Oxidation

Phenolic compounds were extracted from 35 mg of dried plant leave and twig samples using the mild alkaline cupric oxide (CuO) oxidation method developed by Hedges and Ertel [59] as later modified by Goñi and Hedges [60]. Briefly, the samples were transferred into Teflon digestion tubes together with 100 mg of (NH_4_)_2_Fe(SO_4_)_2_∙6H_2_O, 500 mg of CuO, 50 mg of C_6_H_12_O_6,_ 1 mL of ethylvanillin solution (100 mg L^−1^) as internal standard 1 and 15 mL of 2M NaOH and digested at 170 °C for two hours under elevated pressure. Reaction products were cooled overnight and transferred into centrifuge tubes. Then the phenolic compounds were purified by adsorption on C18 columns and desorbed by ethylacetate and concentrated under a stream of nitrogen gas for 30 min. Residue was dissolved in 1 mL phenylacetic acid (PAA), as internal standard 2 to determine the recovery of ethylvanillin before derivatization [61,62]. Finally, the samples were derivatized using 200 μL of BSTFA and 100 μL of pyridine. Oxidation products of phenolic compounds were quantified using a Gas Chromatograph coupled to a Mass Spectrometer (SHIMADZU, GC–MS–QP2010, Kyoto, Japan).

### 3.4. Data Analysis

After recovery correction, the content of each of sixteen phenolic compounds (in g kg^−1^ TOC) was calculated and groups of phenolic derivative of mild alkaline CuO oxidation products were calculated according to Equations (1)–(6).

*p*-hydroxy = 4-Hydroxybenzaldehyde + 4-Hydroxyacetophenone + 4-Hydroxybenzoic acid (1)

Vanillyl (V) = vanillin + 4-hydroxy-3-methoxyacetophenone + vanillic acid (2)

Syringyl (S) = syringaldehyde + 3,5-dimethoxy-4-hydroxyacetophenone + syringic acid(3)

Coumaryl (C) = *p–*coumaric acid + ferulic acid (4)

Benzoic acids = benzoic acid + salicylic acid + phthalic acid(5)

Hydroxy-Benzoic-Acids = 3-hydroxybenzoic acid + 4-hydroxybenzoic acid + 3,5-dihydroxybenzoic acid(6)

#### 3.4.1. Hierarchical Clustering and PCA

Calibrated and normalized data sets of the analytical results were subjected to Principal Component Analysis (PCA, Figure S2) and hierarchical clustering (AHC) using Euclidean measurement of distances and Ward method for linkage calculation to examine taxonomic differences based on phenolic compounds. Z-score normalization method, Z = (X-mean (X))/sd (X), were used to standardize data. Furthermore, descriptive statistics (notched box plot and scatter plot) were performed to identify an unambiguous biomarker using R free software version 3.6.0 [63].

#### 3.4.2. Machine Learning

We examined three different machine learning algorithms (Random forest, Support Vector Machines, and Recursive Partitioning) regarding their accuracy (Figure S1A) and F1 score (Figure S1B) based on different proportions of training and prediction dataset with five replications. Due to the reliable performance of Random forest classification of *Erica* vs. non-*Erica* species, we looked for the most important features in a 2/3 to 1/3 training against prediction setting. To verify the reliability of the used model, cross-validation has been performed (Figure S3).

## 4. Conclusions

Unambiguous identification of chemical proxies using mild alkaline CuO oxidation products of phenols were not simple to apply on plants of the same taxon growing along the SW and NE exposed transects in the Bale Mts. Nonetheless, leaves and twigs of the woody *Erica* shrubs can be distinguished from leaves of the present day dominant herbaceous Poaceae and other lower plants by the relative abundance of coumaryl phenols and benzoic acids. However, as it is known that coumaryl phenols are preferentially degraded in soils, these proxies are not suitable for the evaluation of sites covered in former times by *Erica* (reconstruction of palaeovegetation in soils and sediments). To avoid such ambiguities in future studies, identification of other suitable proxies such as tannin-derived phenols and terpenoids is recommended.

## Figures and Tables

**Table 1:** Summary of the relative abundance of individual phenols profiled in each dominant plant species. Key: ↑ (higher) and ↓ (lower).

Relative Contribution	*Alchemilla*	*Erica*	*Helichrysum*	*Kniphofia*	*Lobelia*	*Poaceae*
Vanillyl phenols					↑	↓
Vanillin						
Vanillic_acid						
_4_hydroxy_3_methoxyacetophenone						
Syringyl phenols	↑				↓	↑
Syringaldehyde						
Syringic_acid						
_3_5_dimethoxy_4_acetophenone						
Coumaryl phenols		↑	↓			↑
p_coumaric_acid						
Ferulic_acid						
p_hydroxy_phenols		↓	↑			
_4_hydroxybenzaldehyde						
_4_hydroxybenzacetophenone						
Benzoic_acids				↑	↑	↓
Benzoic_acid						
Salicylic_acid						
Phthalic_acid						
Hydroxy_benzoic_acids	↑		↓			↑
_3_hydroxybenzoic_acid						
_4_hydroxybenzoic_acid						
_3_5_Dihydroxy_benzoic_acid						

**Table 2:** Phenol Diversity Index.

Species	Diversity Index (H)
*Alchemilla haumannii*	2.28
*Erica* spp.	2.19
*Helichrysum splendidum*	1.63
*Kniphofia foliosa*	2.47
*Lobelia rhynchopetalum*	2.09
*Festuca abyssinica*	2.17

## Supplementary Materials

The following are available online at https://www.mdpi.com/2223-7747/8/7/228/s1, Figure S1: Comparison of Accuracy (A) and F1 Score (B) of Support Vector Machine (SVM, blue), Random Forest (RF, red) and Recursive Partitioning (RP, grey) algorithm based on the level of relative phenols, in relation to the ratio of tested/total number of samples (n = 47) that has been split into test and training datasets, each model has been computed 5 times, the bars indicate the standard derivation between the prediction results of the models; Figure S2: Principal Component Analysis, PCA based on the relative phenol abundance in 47 leaf and twig samples from the Bale Mountains, shown are the first two principal components (PC1 and PC2). Dark red arrows indicate the direction of each vector of feature; Figure S3: Cross-validation of the model used; Table S1: Sum of weighted mean of phenolic compounds of each dominant plant species.
