# Supplementary figures and images for: Phenolic Compounds as Unambiguous Chemical Markers for the Identification of Keystone Plant Species in the Bale Mountains, Ethiopia
Source: Plants (Basel). 2019 Jul 16;8(7):228. doi: 10.3390/plants8070228 (PMC6681346; doi:10.3390/plants8070228)

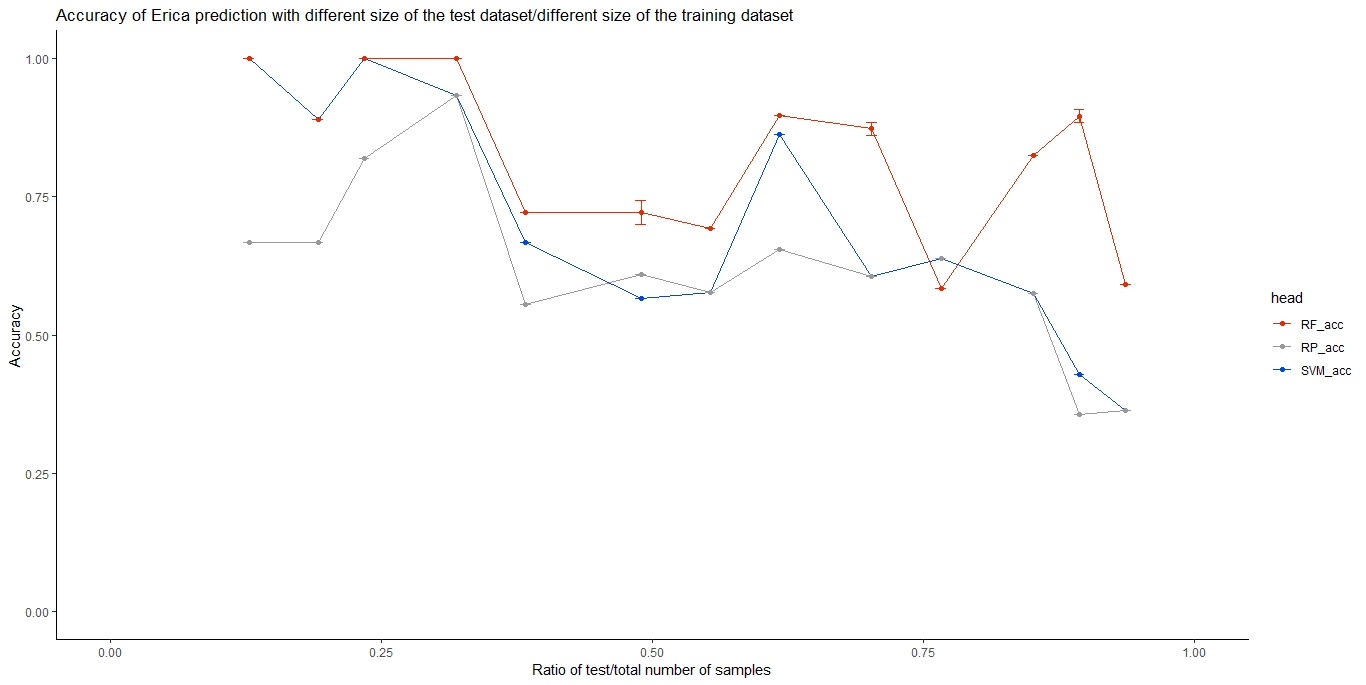

Supplement: Supplementary file 1 [file plants-08-00228-s001.zip › Supplementary Materials/Figure S1A (Accuracy).jpeg]

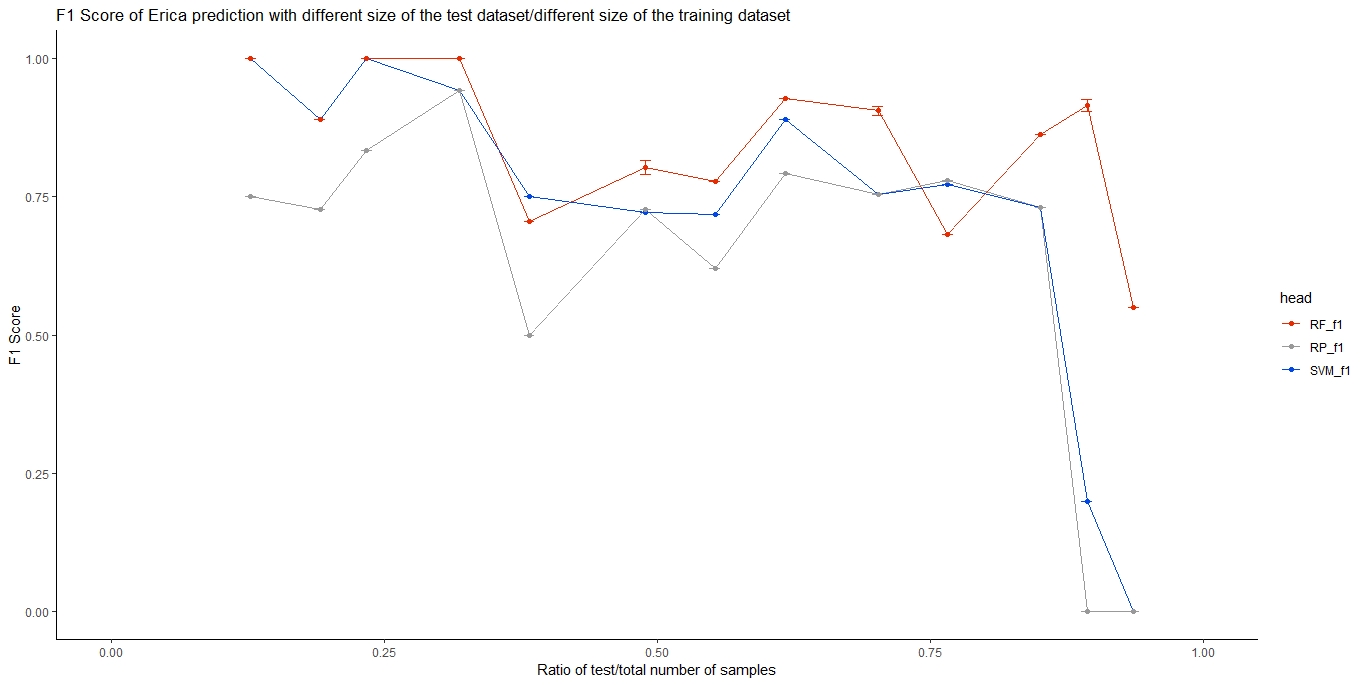

Supplement: Supplementary file 1 [file plants-08-00228-s001.zip › Supplementary Materials/Figure S1B (F1 Score).jpeg]

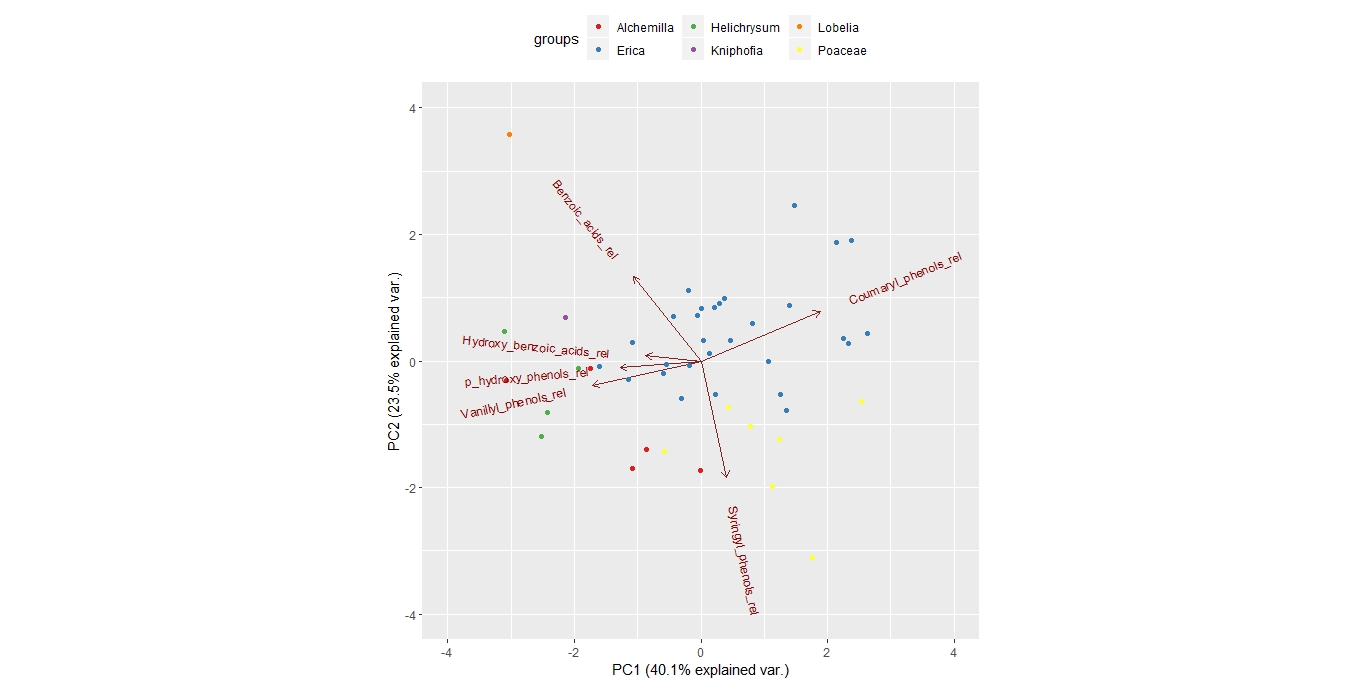

Supplement: Supplementary file 1 [file plants-08-00228-s001.zip › Supplementary Materials/Figure S2_PCA.jpeg]

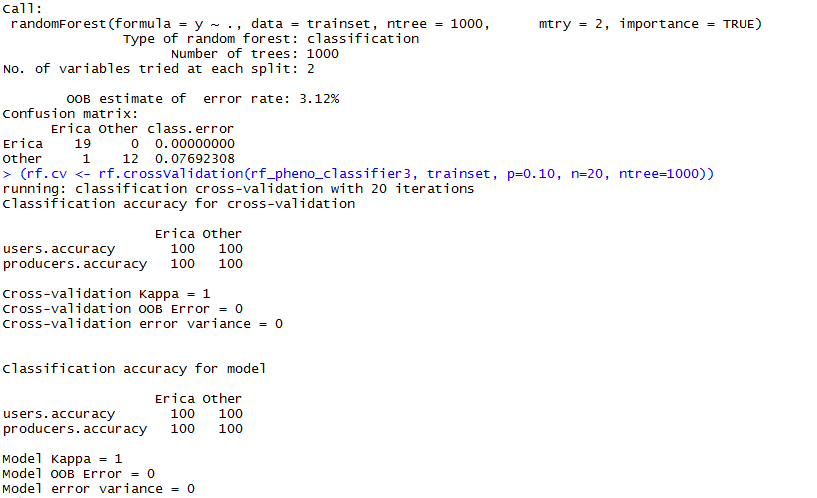

Supplement: Supplementary file 1 [file plants-08-00228-s001.zip › Supplementary Materials/Figure S3_CV.jpeg]
